# Alterations in gut virome are associated with cognitive function and minimal hepatic encephalopathy cross-sectionally and longitudinally in cirrhosis

**DOI:** 10.1080/19490976.2023.2288168

**Published:** 2023-11-27

**Authors:** Thananya Jinato, Masoumeh Sikaroodi, Andrew Fagan, Richard K Sterling, Hannah Lee, Puneet Puri, Brian C Davis, Michael Fuchs, Edith Gavis, Patrick M Gillevet, Jasmohan S Bajaj

**Affiliations:** a Microbiome Analysis Center, George Mason University, Manassas, VA, USA; b Center of Excellence in Hepatitis and Liver Cancer, Department of Biochemistry, Faculty of Medicine, Chulalongkorn University, Bangkok, Thailand; c Division of Gastroenterology, Hepatology and Nutrition, Virginia Commonwealth University and Richmond VA Medical Center, Richmond, VA, USA

**Keywords:** Gut-brain axis, microbiome, hepatic encephalopathy, inhibitory control test, EncephalApp Stroop, psychometric hepatic encephalopathy score

## Abstract

Cognitive dysfunction due to minimal hepatic encephalopathy (MHE) adversely impacts patients with cirrhosis and more precise therapies are needed. Gut-brain axis changes are therapeutic targets, but prior studies have largely focused on bacterial changes. Our aim was to determine linkages between individual cognitive testing results and bacteria with the virome using a cross-sectional and longitudinal approach. We included cross-sectional (*n* = 138) and longitudinal analyses (*n* = 36) of patients with cirrhosis tested using three cognitive modalities, which were psychometric hepatic encephalopathy score (PHES), inhibitory control test (ICT), Stroop, and all three. Stool metagenomics with virome and bacteriome were analyzed studied cross-sectionally and in a subset followed for development/reversal of MHE repeated at 6 months (longitudinally only using PHES). Cross-sectional: We found no significant changes in α/β diversity in viruses or bacteria regardless of cognitive testing. Cognitively impaired patients were more likely to have higher relative abundance of bacteriophages linked with *Streptococcus*, *Faecalibacterium*, and *Lactobacillus*, which were distinct based on modality. These were also linked with cognition on correlation networks. Longitudinally, 27 patients remained stable while 9 changed their MHE status. Similar changes in phages that are linked with *Streptococcus*, *Faecalibacterium*, and *Lactobacillus* were seen. These phages can influence ammonia, lactate, and short-chain fatty acid generation, which are neuro-active. In conclusion, we found linkages between bacteriophages and cognitive function likely due to impact on bacteria that produce neuroactive metabolites cross-sectionally and longitudinally. These findings could help explore bacteriophages as options to influence treatment for MHE in cirrhosis.

## Introduction

There is an alteration in gut-liver-brain axis in patients with cirrhosis and hepatic encephalopathy (HE).^1–3^ These changes are associated with cognitive impairment, aid in the prognostication and prediction of HE-related outcomes and are targets for treatment.^1,2,4^ Cognitive impairment in cirrhosis is mostly due to minimal HE (MHE), the impact of which has been evaluated using several tests such as psychometric hepatic encephalopathy score (PHES), inhibitory control test (ICT), or EncephalApp Stroop.^5^ These tests investigate different aspects of cognition and may reflect varying gut microbial signatures.^6,7^ However, most studies on gut microbiota alterations in cirrhosis and cognitive impairment have primarily focused on bacteria.^6^ Phages and viruses are major modulators of bacterial populations and can also directly affect the human hosts.^8,9^ In prior studies of liver disease and cirrhosis, there are alterations in phage and viral gut microbial populations, which can in turn impact outcomes.^4,8,10^ However, the effect of bacterial-viral linkages on specific cognitive impairments cross-sectionally and longitudinally needs to be investigated. This integrated approach can shed light on the intricate dynamics of the gut ecosystem and help identify novel biomarkers for early detection and intervention in cognitive dysfunction. Our aim was to determine linkages between individual cognitive testing results and bacteria with the virome in a cross-sectional and longitudinal approach in patients with cirrhosis.

## Materials and methods

### Patients

We enrolled outpatients with cirrhosis prospectively after informed consent. Cirrhosis was diagnosed using liver biopsy, transient elastography, evidence of varices, nodular contour of liver or thrombocytopenia in a patient with chronic liver disease or those with documents prior decompensation. Those unclear evidence of cirrhosis, those who were unable to consent, alcohol or illicit drug abuse within 3 months, those on anti-psychotic, anti-seizure, older anti-depressants, or benzodiazepines, those with recent TIPS (<3 months), with recent changes in opioid medications (over the last 3 months) and those with recent (<1 month) hospitalizations were excluded from the study.

**Figure 1.**
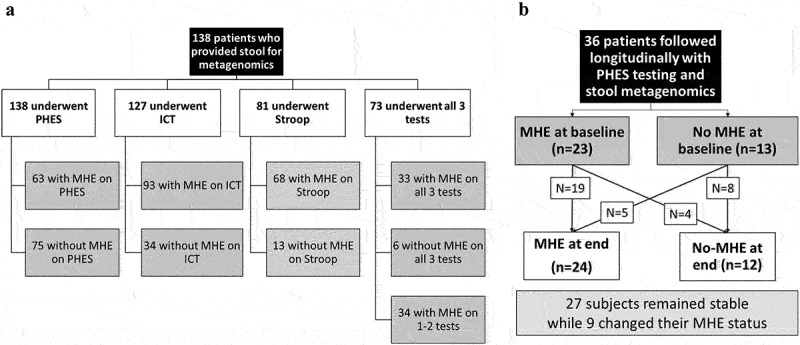
Flow chart of subjects in this study for cross-sectional study (a) and longitudinal study (b).

All patients were first administered the mini-mental status exam (MMSE). If the score was ≥25, we administered cognitive tests using these validated strategies: (a) Psychometric hepatic encephalopathy score (PHES),^11^ (b) Inhibitory Control test (ICT),^12^ and (c) EncephalApp Stroop.^13^ during the same sitting in this order (Supplement). We administered PHES to everyone, while a subset also underwent ICT and EncephalApp Stroop testing. MHE was diagnosed on US-based norms.^14^ Patients with MHE on PHES, Stroop, or ICT individually were studied compared to their counterparts without MHE on these modalities. Finally, those who received all three tests and were normal on all three were compared to those who were abnormal on all modalities (MHE all tests) (Figure 1a).

#### Longitudinal analysis

A subset was followed over time as part of a protocol that evaluated patients every 6 months with PHES testing and stool collection for microbiome (Figure 1b). This follow-up was protocolized, and intercurrent events (hospitalizations, HE episodes, infections, TIPS placement, etc.) were recorded. We excluded those without either MHE testing or stool collection at the follow-up, those who withdrew consent, and those who had received a liver transplant or were lost to follow-up. MHE results on PHES at the end of study were compared to the baseline and MHE dynamics (developed MHE, resolved MHE, remained MHE or remained without MHE) were used to compare clinical and metagenomic changes.

### Stool collection and analysis details

Metagenomic DNA from fecal samples was extracted using the MO BIO PowerFecal DNA Isolation Kit (Qiagen) and stored in our repository at −80°C until the metagenomics analysis.^15^ Samples were processed in an automated, high throughput manner using the QiaCube DNA/RNA Purification System (Qiagen) with bead beating in 0.1 mm glass bead plates. Isolated DNA was quantified and normalized using the Quant-iT Picogreen dsDNA Assay Kit. Shotgun metagenomic libraries were prepared with a procedure adapted from the Nextera Library Prep Kit (Illumina). Libraries were subsequently pooled and assessed using the Agilent Bioanalyzer. Sequencing was performed on either an Illumina NextSeq 550 (1 × 150 bp, NextSeq 500/550 High Output v2 kit) or an Illumina NovaSeq 6000 (1 × 100 bp, NovaSeq 6000 S2 Reagent Kit).

### Metagenomic analysis

Reads were processed and annotated using the BoosterShot in-house pipeline.^15^ Bcl files were converted to fastq format using bcl2fastq (Illumina). Cutadapt.^16^ was used for adapter and quality (final Q-score >20) trimming. Reads shorter than 50 bp were filtered out using cutadapt, and all reads were trimmed to 100 bp prior to downstream alignment and annotation. Quality sequences were then aligned at 97% identity to a curated database (Venti) containing all representative genomes in RefSeq.^17^ for bacteria and additional manually curated strains using the BURST optimal aligner.^18^ Ties in alignment were broken by minimizing the overall number of unique Operational Taxonomic Units (OTUs). For taxonomic assignment, each input sequence was assigned the lowest common ancestor which was consistent across at least 80% of all reference sequences tied for best hit. Counts were normalized to the species-level average genome length. OTUs accounting for less than one millionth of all species-level genomic markers were discarded, as well as those with either less than 0.01% of their unique genome or less than 1% of the whole genome covered by reads in any sample.

Lastly, viral taxonomic classification of reads was performed using VirMap.^19^ (using default parameters and the following flags: –useMegahit – useBbnorm) in conjunction with Diversigen’s custom viral database containing all representative genomes in RefSeq.^17^ VirMap uses a combination of nucleotide and amino acid mapping (via BBMAP and DIAMOND) and do-novo assembly (using MEGAHIT and/or Tadpole) strategies to generate and annotate viral contigs, which performs well even on viral genomes with fairly low coverage.^19^ In accordance with VirMap’s default parameters, viral taxa with less than 1000 bits of alignment information were removed. Phage-only tables were created by removing all non-phage viral taxa. Phage sharing the same hierarchical classification was collapsed at the genus level.

### Bioinformatics analysis

The calculation of alpha diversity metrics (diversity of microbiome within specific samples), including Shannon, Chao1, and Simpson indices, was performed to assess the diversity within a specific sample using the vegan R package.^20^ This analysis was conducted on count tables that were rarefied to 40,000 reads per sample. The between-sample beta diversity, which calculates the distance-to-centroid to measure dissimilarity between different samples, was calculated using Bray-Curtis distance with the vegan package. Beta-diversity: Principal coordinate analysis (PCoA) plots were constructed using Bray-Curtis distance to assess beta-diversity (differences in microbial diversity between groups). Individual taxa differences were analyzed using linear discriminant function effect size (LEFSe) analysis.^21^ We first analyzed patients who were PHES positive compared to PHES negative in the entire population, similarly for ICT and then for Stroop. Lastly, we analyzed changes between those who had MHE on all three tests compared to those who were negative on all three tests. Significance was tested using a p-value cutoff of 0.05.

### Correlation network analysis

We analyzed correlations between bacterial species and phage genera in patients in the cross-sectional study and stable and unstable changes over time using published R techniques.^22^ Only data that were *r* > 0.6/r-<0.6 and *p* < 0.05 were filtered and visualized using Cytoscape. Correlation network characteristics were compared between groups within these cross-sectional and longitudinal studies.

## Results

### Cross-sectional study

We included 138 patients with cirrhosis who all underwent PHES testing and stool collection. Of the 138, 127 also underwent ICT, 81 underwent EncephalApp Stroop, and 73 underwent all three tests (8 patients who were given EncephalApp Stroop did not get ICT).

The prevalence of MHE varied across the tests (Figure 1a). Of the 138 subjects, 52% had prior HE on lactulose or rifaximin. The leading etiologies of cirrhosis were alcohol (*n* = 49), hepatitis C (*n* = 50), both (*n* = 23), and the rest were metabolic dysfunction-associated steatotic liver disease (MASLD). Comparisons between those with/without MHE based on the three groups are shown in Table 1. Patients who have MHE on PHES were older and more likely to be men but remaining liver disease severity and etiology, prior HE and medications were similar compared to those without MHE on PHES. When ICT was considered, prior HE and lactulose use were higher in MHE-ICT versus the rest, but remaining demographics, cirrhosis details, and medications were similar compared to patients without MHE in ICT. In the subgroup who were given EncephalApp, patients who had MHE were older and more likely to be men. Remaining parameters (Table 1) were similar.

**Table 1.** Clinical characteristics and alpha diversity metrics between groups cross-sectionally.

Cross-sectional	MHE-PHES (*n* = 138)		MHE-ICT (*n* = 127)		MHE-Stroop (*n* = 81)		MHE All tests	
Mean±SD	No MHE (*n* = 75)	MHE (*n* = 63)	No MHE (*n* = 34)	MHE (*n* = 93)	No MHE (*n* = 13)	MHE (*n* = 68)	No MHE (*n* = 6)	MHE (*n* = 33)
Age (years)	**58.3 ± 6.6**	62.8 ± 6.2*	58.2 ± 7.1	60.8 ± 6.7	**57.0 ± 6.8**	63.9 ± 6.0*	60.78 ± 6.22	65.01 ± 4.49
Male sex	**46**	56*	24	70	**6**	57*	3	9
Race (White/Black/Hispanic)	53/18/4	42/20/1	24/7/3	65/26/3	13*/0/0	**44/22/2**	5/1/0	19/13/1
MELD	10.9 ± 3.8	10.7 ± 3.5	10.6 ± 3.7	11.1 ± 3.6	11.7 ± 4.4	10.9 ± 3.5	11.5 ± 6.16	10.52 ± 3.56
Etiology (HCV/alcohol/HCV and alcohol/Others	28/24/13/10	22/25/10/6	13/11/6/4	33/35/13/12	5/4/2/2	24/20/12/11	3/1/2/0	15/9/7/2
PPI use	40	40	15	55	6	42	3	19
Lactulose	40	31	**10**	56*	8	40	2	18
Rifaximin	27	24	6	41	6	36	2	16
Prior HE	40	32	**10**	56*	8	40	2	18
**Bacterial diversity**								
Shannon	2.8 ± 0.6	2.7 ± 0.8	2.7 ± 0.6	2.8 ± 0.7	2.7 ± 0.5	2.6 ± 0.7	2.8 ± 0.3	2.5 ± 0.8
Simpson	0.9 ± 0.1	0.8 ± 0.2	0.9 ± 0.1	0.9 ± 0.1	0.9 ± 0.1	0.8 ± 0.2	0.9 ± 0.01	0.8 ± 0.2
Chao1	261.1 ± 329.7	294.9 ± 354.5	206.0 ± 280.2	326.7 ± 368.9	243.9 ± 314.6	190.1 ± 269.0	205.3 ± 324.0	213.6 ± 287.9
**Viral diversity**								
Shannon	1.3 ± 0.6	1.3 ± 0.6	1.3 ± 0.7	1.3 ± 0.5	1.4 ± 0.7	1.3 ± 0.5	1.4 ± 0.8	1.5 ± 0.5
Simpson	0.6 ± 0.2	0.5 ± 0.2	0.6 ± 0.2	0.6 ± 0.2	0.6 ± 0.3	0.5 ± 0.2	0.6 ± 0.3	0.5 ± 0.2
Chao1	**15.3 ± 8.9**	19.8 ± 9.2*	15.6 ± 11.0	18.1 ± 8.3	14.6 ± 8.2	18.7 ± 9.3	12.5 ± 8.1	19.1 ± 8.3

MHE: minimal hepatic encephalopathy, SBP: spontaneous bacterial peritonitis, MELD: model for end-stage liver disease score, PPI: proton pump inhibitors, HCV: hepatitis C, SD; standard deviation. PHES: psychometric hepatic encephalopathy score, ICT: inhibitory control test, MHE: minimal hepatic encephalopathy, all tests: No-MHE is normal on all three, MHE is abnormal on all three, PHES: psychometric hepatic encephalopathy score.

* and bold font indicate significant differences (p<0.05) in the comparisons that are in that font.

### Cross-sectional gut microbiota changes with cognitive testing

*α-diversity*: In those with PHES abnormalities, while Shannon and Simpson indices were similar for both viruses and bacteria, the chao1 was higher with respect to viral but similar between MHE vs not for bacteria. MHE according to ICT and Stroop showed similar bacterial and viral measures of α-diversity. There was a higher viral chao1 in those who had MHE on all three tests compared to those who were normal on all three, while other viral and all bacterial α-diversity indices were statistically similar.

**Figure 2.**
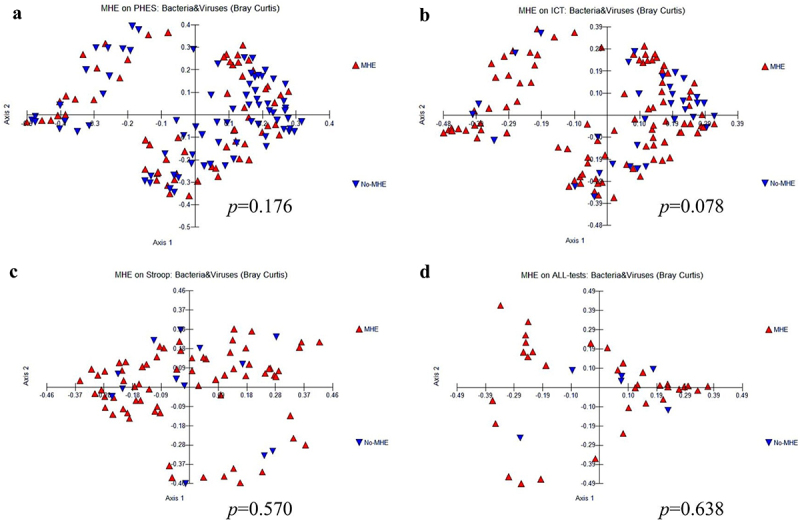
β diversity combined both bacteria and viruses comparisons between MHE and No-MHE. (a) PHES, (b) ICT, (c) Stroop, and (d) all tests.

*β-diversity*: However, the PERMANOVA test showed no significant changes in beta-diversity between individuals with minimal HE (MHE) and those without it, concerning both bacteria and viruses, as well as their combined taxonomy. This analysis was performed based on data obtained from the PHES (Figure 2a), ICT (Figure 2b), and Stroop (Figure 2c), collectively evaluated across all three tests (Figure 2d).

*Individual taxonomy changes*: In Table 2, patients without MHE on PHES demonstrated higher commensal (*Lachnospiraceae*, *Ruminococcus*, *Anaerostipes*, and *Eubacterium* spp), while potential pathobionts (*Escherichia, Klebsiella*) and lactate producers (*Streptococcus* and *Lactobacillus* spp) were higher in those with MHE. Phages associated with *Lactobacillus* especially against *L. fermentum*, *Streptococcus* satellite phage, and *Bacteroides* phage as well as those linked with Enterobacteriaceae (*gokushovirus*, *Escherichia* virus phiX174) were higher in those with MHE, while Siphoviridae and crAssphages showed the opposite pattern (Figure S1). On ICT, no-MHE patients had higher *Ruminococcus*, Lachnospiraceae, *Lactobacillus*, and *Bifidobacterium* spp and among viruses, higher *Escherichia* phages and *Streptococcus* phage spp (Figure S1). With Stroop, MHE patients had higher *Escherichia* spp and lower *Oscillibacter* and *Bacteroides*. No-MHE patients showed greater phages associated with *Lactococcus, Enterococcus, Lactobacillus, Klebsiella*, and *Streptococcus* (Figure S1). When all three MHE strategies showed impairment, the pattern of bacterial and viral taxa was similar to that seen with Stroop (Figure S1).

**Table 2.** LEfSe results across different cognitive testing strategies cross-sectionally.

MHE testing using PHES		MHE testing using ICT		MHE testing using EncephalApp Stroop		MHE on all 3 tests	
No MHE (*n* = 75)	MHE (*n* = 63)	No MHE (*n* = 33)	MHE (*n* = 93)	No MHE (*n* = 13)	MHE (*n* = 68)	No MHE on all (*n* = 6)	MHE on all (*n* = 33)
*Bifidobacterium pseudo-catenulatum* *Eubacterium* *hallii* *Alistipes_sp_AP11* *Subdoligranulumsp_4_3_54A2FAA* *Ruminococcus* *callidus* *Ruminococcus_sp_5_1_39BFAA*	*Lactobacillus casei paracasei* *Bifidobacterium dentium* *Bifidobacterium breve* *Lactobacillus fermentum* *Klebsiella pneumoniae*	*Ruminococcus* *gnavus* *Lachnospiraceae bacterium_5_1_63FAA* *Lactobacillus Oris* *Bifidobacterium longum*	none	*Bacteroides sp_1_1_6* *Oscillibacter sp KLE 1745*	*Paraprevotella unclassified* *Barnesiella intestinihominis* *Escherichia* *unclassified* *Escherichia_coli*	*Acidaminococcus_sp_HPA0509* *Bacteroides coprocola* *Bacteroides_sp 1_1_6* *Oscillibacter sp KLE_1745* *Faecalibacterium prausnitzii* *Clostridium sp KLE 1755* *Lachnospiraceae bacterium_5_1_57FAA* *Eggerthella unclassified* *Clostridium nexile*	none
*Siphoviridae* *CrAssphage sp_152*	*Human gut gokushovirus* *Escherichia virus phiX174* *Lactobacillus phage LfeInf* *Lactobacillus phage Lfe Sau* *Microviridae sp_* *Bacteroides phage crAss001* *Azobacteroides phage ProJPt_Bp1* *Streptococcus satellite phage Javan 319*	*Escherichia phage EG1* *CrAssphage sp* *_152* *Streptococcus phage Javan 210* *Streptococcus phage P0091*	none	*Enterococcus virus AUEF3* *Lactobacillus phage phiPYB5* *Enterococcus virus EfaCPT1* *Streptococcus phage CHPC1148* *Enterococcus phage* *vB_EfaS_Ef6_4* *Lactococcus phage 79,201* *Lactococcus Phage 51,701* *Streptococcus satellite phage Javan289* *Pseudomonas virus Andromeda* *Streptococcus virus Cp7* *Streptococcus phage CHPC919* *Klebsiella virus GML_KpCol1* *Lactobacillus phage phiJB* *Salmonella virus SPN 3US* *Escherichia virus VpaE1* *Thermus phage G20c* *Streptococcus phage CHPC877* *Pseudomonas virus tabernarius*	none	*Enterococcus virus AUEF3* *Lactococcus virus Sl4* *Enterococcus virus EfaCPT1* *Enterococcus phage vB_EfaS_Ef6_4* *Lactococcus phage79201* *Streptococcus virusCp7* *Klebsiella virus GML_KpCol1* *Escherichia virus VpaE1* *Streptococcus satellite phage Javan312*	none

### Cross-sectional correlation network characteristics

**Figure 3.**
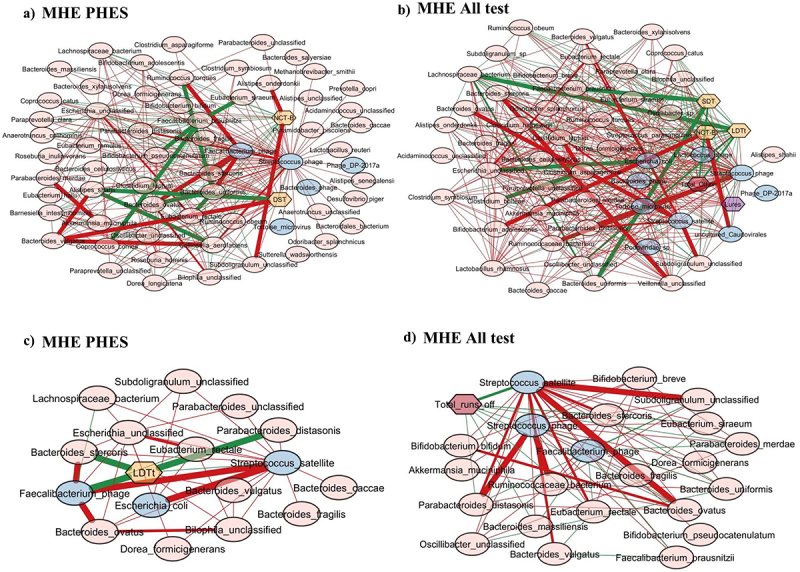
Cross-sectional correlation network shown centered around *Streptococcus* phage (a) MHE PHES, (b) MHE all test and centered around *Streptococcus satellite* phage (c) MHE PHES, (d) MHE all tests. Pink nodes: bacterial genera, blue nodes: viral genera, peach nodes: PHES subtests. Purple nodes: ICT values and Dark Pink nodes: Stroop values. Red lines: negative correlation and green lines: positive correlation.

**Figure 4.**
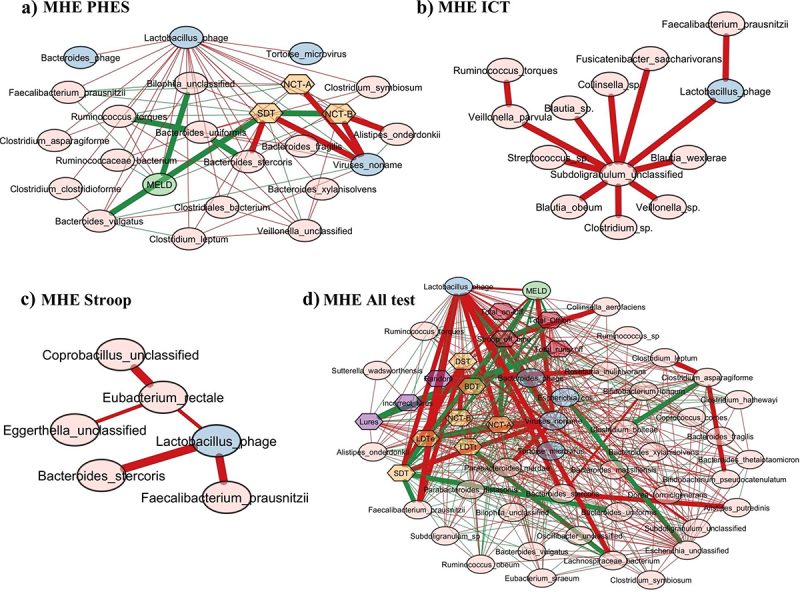
Cross-sectional correlation network shown centered around *Lactobacillus* phage (a) MHE PHES, (b) MHEICT, (c) MHE Stroop, and (d) MHE all test. Pink nodes: bacterial genera, blue nodes: viral genera, peach nodes: PHES subtests. Purple nodes: ICT values and Dark Pink nodes: Stroop values. Red lines: negative correlation and green lines: positive correlation.

When *Streptococcus* phage was evaluated, in MHEPHES, it was negatively linked with number connection test B (NCT-B) and positively with digit symbol test (DST), which indicates better cognitive performance. *Streptococcus* phage was negatively linked with *Faecalibacterium prausnitzii* and *Faecalibacterium* phage (Figures 3a, 3b). Similar linkages with MHEAll tests were seen with negative correlation with *Faecalibacterium prausnitzii*, symbol digit test (SDT), line drawing test (LDTt) and positive linkage with NCT-B Lures, NCT-B, Total_Off-on. *Streptococcus* satellite phages were negatively linked with *Faecalibacterium* phage and positively with *Streptococcus* phage in MHEPHES and MHEAll tests (Figures 3c, 3d). In MHEPHES, *Lactobacillus* phage was linked negatively with model for end-stage liver disease (MELD), number connection test A/B (NCT-A/B), SDT as well as with *Faecalibacterium prausnitzii*. This negative linkage with *Faecalibacterium prausnitzii* was also found in MHEICT, MHEStroop, and MHEAll test correlation networks. In MHEAll test patients, *Lactobacillus* phages were also negatively correlated with MELD, components of PHES and other tests in whom a high score indicates poor performance (NCT-A/B SDT, line tracing test: LTTe, Stroop times) and positively with those in which a high score indicates good performance (block-design test: BDT, DST) apart from lures. No linkages were seen in MHEStroop and MHEICT (Figure 4).

**Figure 5.**
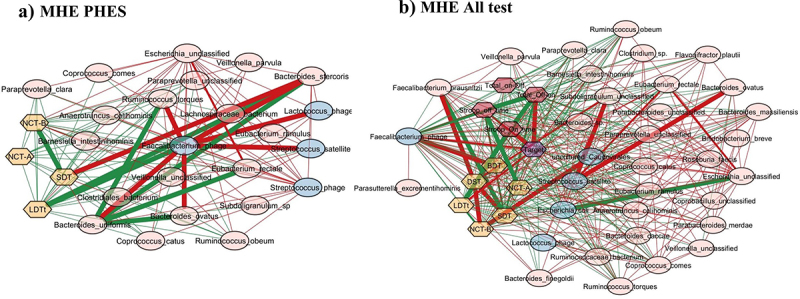
Cross-sectional correlation network shown centered around *Faecalibacterium* phage (a) MHE PHES, (b) MHE all test. Pink nodes: bacterial genera, blue nodes: viral genera, peach nodes: PHES subtests. Purple nodes: ICT values and Dark Pink nodes: Stroop values. Red lines: negative correlation and green lines: positive correlation.

When subnetworks around *Faecalibacterium* phage were analyzed, we found positive linkage with tests in whom a high score indicates poor cognition (LDTt, NCT-A, NCT-B, SDT) in MHEPHES. In MHEAll test patients, *Faecalibacterium* phage was negatively linked with BDT, DST, Target, where a high score indicates poor performance and was positively linked with tests where the reverse is seen (NCT-A/B, SDT, LTTt, Stroop time measures) and with *Faecalibacterium prausnitzii*. No linkages were seen in MHEStroop and MHEICT (Figure 5).

### Longitudinal study

Thirty-six patients, mostly men (*n* = 32) with a mean age of 63 ± 6.3 years, were who had PHES testing done and were enrolled in this portion of the study (Figure 1b). The enrollment MELD score was 11.2 ± 3.5, and 31 had prior HE on lactulose (*n* = 26) and rifaximin (*n* = 24). PPI use was seen in 26 patients. They were followed for 9 ± 3 months when PHES and stool collection was repeated.

*Clinical status change*: Over time three patients in MHE group developed ascites, three in the MHE group developed an episode of overt HE for which they were started on lactulose, while none of them developed variceal bleeding. Twenty-three patients had MHE at baseline, of which 19 remained MHE while 4 resolved. Thirteen patients were without MHE at baseline, of which eight remained in this state, while five developed new-onset MHE. Apart from those who developed MHE, most patients in the longitudinal cohort already had prior HE. The patients who developed new-onset MHE also developed ascites, SBP and new-onset HE with lactulose and/or rifaximin initiation. In those who resolved MHE, there were no new complications over time. No changes in alpha diversity measures were seen across the three groups (stable MHE, stable no-MHE, developed MHE), but there was a decrease in viral and bacterial Chao1 in those who resolved MHE. Due to the small N, this did not reach significance.

**Figure 6.**
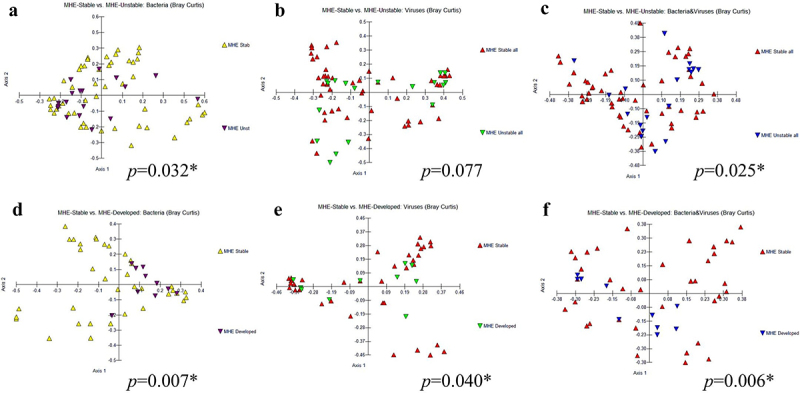
β diversity of longitudinal analysis between stable versus unstable in bacteria (a), viruses (b), and combined both bacteria and viruses (c) and the comparisons between stable MHE versus developed MHE in bacteria (d), viruses (e), and combined both bacteria and viruses (f).

*Stable versus unstable comparison*: Of the 28 people who were stable throughout the study (MHE remained MHE, No-MHE remained No-MHE), there was no significant change in disease severity as well, along with statistically similar viral and bacterial alpha diversity (Table 3 and S2). In the nine pairs that either developed MHE or resolved it, there was a greater clinical change over time with higher HE episodes with greater lactulose and rifaximin use. There were lower Chao1 viral and bacterial indices at the end of follow-up without any significant changes on other indices. Regarding β-diversity in bacteria, the PERMANOVA test revealed significant differences across the groups (*p* = 0.032) (Figure 6a). However, for viruses alone, there was no statistically significant difference (*p* = 0.077) (Figure 6b). Interestingly, when considering both bacteria and viruses together, a significant difference emerged (*p* = 0.025) (Figure 6c). Higher relative abundance of *Lactobacillus* and *Bifidobacterium* spp and lower relative abundance of a mixture of potential pathobionts (*Enterobacter, Haemophilus*) and SCFA producers were seen in the unstable group. Phages centered around *Streptococcus* were the predominantly higher taxa in the unstable group, although *Escherichia, Lactococcus*, and *Faecalibacterium* phages were also seen (Figure S2).

**Table 3.** Comparison between patients whose MHE remained stable and those in whom MHE developed on follow-up.

	MHE Stable (*n* = 19 pairs)		MHE Developed (*n* = 5 pairs)		LEFSe	MHE stable	MHE developed	
Mean±SD	Pre	Post	Pre	Post	**Bacteria significant** **LDA score > 3**	*Bifidobacterium pseudocatenulatum* *Lactobacillus casei paracasei* *Bifidobacterium dentium* *Bifidobacterium adolescentis* *Bifidobacterium breve* *Klebsiella pneumoniae* *Lactobacillus rhamnosus*		*Phascolarctobacterium succinatutens* *Bacteroides uniformis* *Ruminococcus obeum* *Eubacterium biforme* *Roseburia hominis* *Bacteroides faecis* *Roseburia inulinivorans* *Blautia hydrogenotrophica* *Prevotella stercorea* *Ruminococcus sp_5_1_39BFAA* *Eggerthella unclassified* *Dorea longicatena* *Lachnospiraceae_bacterium_7_1_58FAA* *Eubacterium eligens*
MELD score	12.6 ± 3.1	12.4 ± 3.4	12.3 ± 4.6	13.0 ± 5.4				
PPI	17	17	2	2				
Lactulose	19	20	1	3				
Rifaximin	18	19	0	2				
Prior HE	19	20	1	4				
Ascites worsening	-	0	-	3				
Overt HE episodes	-	0	-	3				
**Bacterial diversity**								
Shannon	2.2 ± 0.9	2.3 ± 0.6	2.8 ± 0.2	2.8 ± 0.3	**Viruses significant** **LDA score > 2**	*Lactobacillus phage LfeSau*		*Faecalibacterium phage FP_Brigit* *Streptococcus phageYMC_2011* *Streptococcus phage Javan268*
Simpson	0.7 ± 0.2	0.8 ± 0.1	0.9 ± 0.00	0.9 ± 0.00				
Chao1	75.1 ± 17.2	76.3 ± 16.5	90.0 ± 19.6	81.6 ± 18.6				
**Viral diversity**								
Shannon	1.2 ± 0.5	1.1 ± 0.6	1.1 ± 0.6	1.1 ± 0.7				
Simpson	0.5 ± 0.2	0.5 ± 0.2	0.5 ± 0.2	0.5 ± 0.3				
Chao1	17.8 ± 7.5	15.3 ± 6.6	14.0 ± 14.6	15.8 ± 9.2				

*Stable MHE versus those who developed MHE*: In terms of β-diversity, the PERMANOVA test showed significant differences among the groups for bacteria (*p* = 0.007), viruses (*p* = 0.043), and the combined analysis of both bacteria and viruses (*p* = 0.006) (Figure 6d-f). We found a significantly higher relative abundance of *Lactobacillus* and *Bifidobacterium* spp and higher *Klebsiella pneumoniae* versus those who ultimately developed MHE. In patients who ultimately developed MHE, there was a higher relative abundance of SCFA-producing taxa at baseline, along with *Bacteroides, Prevotella*, and *Eggerthella* spp. These were associated with higher relative abundance of phages associated with *Faecalibacterium* and *Streptococcus*, while the reverse was seen with the *Lactobacillus* phage LfeSau (Figure S2).

*Stable No-MHE vs resolved MHE*: Resolved MHE patients were more likely to have higher relative abundance of Streptococcus and Escherichia-associated phages as well as greater Escherichia, Klebsiella, and Bifidobacterium spp. On the other hand, resolved MHE patients had lower *Streptococcus salivarius, Ruminococcus obeum*, and *Bacteroides xylanisolvens*

## Discussion

Our data show that virus-bacteria linkages in patients with cirrhosis are associated with changes in cognitive function both cross-sectionally and longitudinally. Bacteriophages linked with *Streptococcus*, *Faecalibacterium*, and *Lactobacillus* are associated with MHE at baseline and in those followed over time for MHE resolution and those who developed new-onset MHE. Viral-bacterial correlation characteristics with cognitive function are also different between MHE and no-MHE and are most prominent when PHES was considered the testing strategy for MHE.

We extended prior studies by evaluating the role of viruses, including bacteriophages, in modulation of the gut bacteria with cognition in a cross-sectional and longitudinal manner using three testing modalities. We confirmed that patients with cognitive impairment have greater potential bacterial pathobionts and lower SCFA producers.^6^ along with *Lactobacillus* spp, and these patterns differed between testing modalities.^6,10^ Phages focused on *Lactobacillus*, *Streptococcus*, and *Escherichia* were higher, while crAssphages and Siphoviridae spp were lower in patients with MHEPHES. This contrasted with MHEStroop and MHEICT where *Streptococcus*, *Enterococcus*, and *Escherichia* phages were higher in those without MHE. Moreover, there were also differences in the linkages between bacteria and phages depending on the test used. These findings underline the relative specificity of the phage-bacterial interactions for each testing modality and are unlikely to be an association with the liver disease severity itself.

The higher Siphoviridae and lower Myoviridae constituents in MHEPHES patients extend a recent study of phages affecting executive function and cognition in healthy individuals to patients with cirrhosis.^23,24^ This includes the trail-making test-B or NCT-B, which is an essential part of PHES and was also individually linked with constituents of these families. Most phages that target lactic acid bacteria belong to Caudovirales, which could explain the correlation of *Streptococcus* and *Lactobacillus* phages with better cognition.^23,24^ Similarly, in those who had better response inhibition and inhibitory control (No-MHE in ICT or Stroop), we found higher constituents of the erstwhile Caudovirales order that are linked with *Enterococcus* spp.^25^ While the reasons behind these are unclear, serotonin production in *Enterococcus* could lead to impaired response inhibition, which is a major part of the cognitive domains tested by ICT and Stroop but not PHES. In our prior study with only bacteria, we found that *Enterococcus* spp was lower in MHEICT uniquely, whereas here we found that *Enterococcus* phages were higher in the same group. This is intriguing because *Enterococcus* spp produces serotonin that promotes inhibitory control and lowers impulsivity.^26,27^ While we did not perform incubation experiments, lytic phages against *Enterococcus* could reduce that function or relative abundance of their hosts in MHEICT and MHEStroop. Our experience sets the stage for further investigation to determine if modulation using phages can influence cognitive function in cirrhosis through selective targeting of bacteria that produce neurotransmitters.

*Streptococcus* spp are important modulators of the gut-liver-brain axis in cirrhosis due to their ability to express urease that is ammoniagenic and have been associated with MHE.^28^ In a prior study, exposure to rifaximin, which improves cognitive function in cirrhosis, resulted in reduction in complexity of *Streptococcus* phage/*Streptococcus* satellite phage and *Streptococcus* correlations.^10^ This was also found in resolved MHE patients where *Streptococcus* phages were higher while the urease-expressing *S. salivarius* was lower. *Faecalibacterium* spp is associated with better gut barrier function and production of SCFA and is typically higher in patients with good cognitive function in cirrhosis.^29,30^ Therefore, the higher relative abundance of lytic *Faecalibacterium* phages in cognitively impaired patients and correlation of these phages with poor cognitive testing could be due to their lytic action against *Faecalibacterium* .^31^ On the other hand, *Lactobacillus* phages had a complicated relationship with bacteria and cognitive function longitudinally and cross-sectionally.^32^. *Lactobacillus* spp often increases as a result of HE therapy, and lactate production is usually associated with benefit; however, with lactate overproduction it can impair intestinal and brain function.^33,34^ Therefore, the complex modulation of *Lactobacillus* with phages could be of benefit and change with therapy in HE.

Given that we had to use a more lenient cut-off to detect phages compared to bacteria on LEFse and biological plausibility, it is likely that phages act through bacterial modulation rather than directly interacting with the host.^35,36^ The implications of these findings are that phages could help manipulate brain function in cirrhosis through precise modulation of bacteria over and above the current therapies, which could help us target those with residual cognitive deficits.^37^ Sterile filtrates and fecal microbiota transplants have the potential to change viromes and bacteriomes in cirrhosis, but phages can be more precise and potentially target either ammonia producers, reduce overabundance of lactate, and help reduce inflammation in future studies.^38–40^

The strengths of our study are well-characterized patients with multiple types of cognitive impairment spanning several domains, longitudinal follow-up with dynamic cognitive testing, and a detailed phenotype-bacteriome-virome linkage. Our study is limited by the relatively small number of patients studied longitudinally, the metagenomic rather than virome-like particle assessment of viruses, unclear mechanism(s) behind these changes, and was also limited by the lack of pre/post testing after defined therapies. Phage-bacterial linkages could also reflect the impact of therapies rather than the underlying disease, although the different patterns of phage-bacterial correlations seen with specific MHE modalities and longitudinal analyses hint otherwise. Similar to prior studies, we found that prior HE patients, despite being on lactulose or rifaximin, continued to exhibit persistent cognitive impairment.^37^ We used different cutoffs for phages and bacteria because the relative abundance changes in bacteria were much more robust than phages. We also did not perform direct incubation experiments of phages with bacterial targets.

We conclude that in addition to bacteria, changes in viruses such as phages linked with *Streptococcus*, *Faecalibacterium*, and *Lactobacillus* are associated with cognitive impairment cross-sectionally and differ based on the cognitive domain tested. Bacteriophages are also associated with dynamics of development, stability, and resolution of cognitive impairment over time. The modulation of bacteria that produce neuroactive metabolites such as ammonia, short-chain fatty acids, and lactate through phages could impact cognitive domains in cirrhosis over and above the current standard of care therapies. The findings from this research may provide valuable insights into novel therapeutic avenues for managing cognitive impairments in cirrhosis, thus enhancing the overall quality of life for affected patients.

## Supplementary Material

Supplement virome cognition clean.docx

Gut Microbes Supplementary Figures_110823.docx

## Data Availability

Given restrictions placed by our institutional review boards at the time of data collection and consent, individual data are not available.

## Abbreviations

BDT, block-design test; DST, digit symbol test; ICT, inhibitory control test; LEfSe, Linear discriminant analysis Effect Size; LTT, line tracing test; MELD, model for end-stage liver disease; MHE, minimal hepatic encephalopathy; MMSE, mini-mental status exam; NCT-A, number connection test A; NCT-B, number connection test B; OUT, operational taxonomic unit; PERMANOVA, permutational multivariate analysis of variance; PHES, psychometric hepatic encephalopathy score; SCFA, short-chain fatty acids; SDT, serial dotting test; TIPS, transjugular intrahepatic portosystemic shunt
