# Supplementary material for: Alterations in gut virome are associated with cognitive function and minimal hepatic encephalopathy cross-sectionally and longitudinally in cirrhosis
Source: Gut Microbes. 2023 Nov 27;15(2):2288168. doi: 10.1080/19490976.2023.2288168 (PMC10730154; doi:10.1080/19490976.2023.2288168)
Supplement: Supplement virome cognition clean.docx [file KGMI_A_2288168_SM6798.docx]

Alterations in Gut Virome are Associated with Cognitive Function Cross-Sectionally and Longitudinally in Cirrhosis

# Supplementary Methods:

## Testing strategies for Minimal Hepatic Encephalopathy (MHE)

PHES details^1^: This validated five test paper-pencil battery which tests visuo-motor coordination, psychomotor speed, and reaction time is considered the gold standard of MHE testing^2^. This battery consists of number connection test-A (NCT-A), number connection test B (NCT-B), digit symbol test (DS), serial dotting test (SD), and line tracing test (LTT has two components: time and errors). Among these tests, a high raw score on digit symbol and low time for completion or errors in the remaining tests indicate good cognition. Based on population control values, the standard deviations are calculated for each sub-test, and the total is added to give one value^3^. A high score on the total PHES score indicates better performance.

Inhibitory control test^4^:  This computerized test involves the presentation of several letters at 500-ms intervals which have letters X and Y interspersed within them. The subject being tested is supposed to respond to every X and Y during the initial part of the training run, in order to establish the prepotent response. In the second part of training, the subject is instructed only to respond when X and Y are alternating (called targets) and inhibit from response when X and Y do not alternate (called lures). After the subject is trained, 6 test runs, which consist of 40 lures, 212 targets, and 1728 random letters are presented at the end of which lure and target response rates are automatically calculated. Lower lure response and higher targets indicate better cognitive performance.

EncephalApp Stroop testing^1, 5^: This App has two components: “off” and “on” state which were administered after two training runs. In the easier “off” state, the subject views a neutral stimulus, pound signs (###) presented in red, green, or blue, one at a time. They need to respond as quickly as possible by touching the matching color that are shown at the bottom of the screen. The “on” state shows incongruent stimuli where the subject needs to accurately touch the color of the word presented, which is discordant. E.g., the word “RED” is displayed in blue color and the correct response is blue not red. One run consists of 10 presentations that are successfully completed, and we needed 5 correct runs to be completed. Outcome values include the total time taken for the run as well as the individual responses. If the subject makes a mistake, i.e., presses a wrong color, the run stops and has to start again. Therefore, the number of runs required to make five correct runs also indicates the number of mistakes. We continue both off and on states five correct runs are achieved. The specific outcomes at the end of the EncephalApp app are: (a) total time for five correct runs in the “off” state (OffTime); (b) number of runs needed to complete the five correct “off” runs; (c) total time for five correct runs in the “on” state (OnTime); and (d) number of runs needed to complete the five correct “on” runs. The test of cognitive processes controlling for psychomotor speed was obtained by subtracting the OffTime from the OnTime, and this was performed for all groups. OffTime+OnTime has been found to be the best discriminator between subjects with and without MHE.

MHE on PHES, EncephalApp Stroop and ICT were based on norms created for the Virginia population^1^.

Table S1: Beta-Diversity PERMANOVA differences cross-sectionally and longitudinally

| Organism type | Cross-sectional | PERMANOVA  P value | Longitudinal | PERMANOVA  P value |
| --- | --- | --- | --- | --- |
| Bacteria | MHE-PHES | 0.23 | MHE Stable vs. MHE Unstable | 0.032* |
|  | MHE-ICT | 0.07 |  |  |
|  | MHE-Stroop | 0.48 | MHE Stable vs. MHE Developed | 0.007* |
|  | MHE-ALL-tests | 0.54 |  |  |
| Viruses | MHE-PHES | 0.14 | MHE Stable vs. MHE Unstable | 0.087 |
|  | MHE-ICT | 0.13 |  |  |
|  | MHE-Stroop | 0.61 | MHE Stable vs. MHE Developed | 0.04* |
|  | MHE-ALL-tests | 0.70 |  |  |
| Bacteria and Viruses | MHE-PHES | 0.18 | MHE Stable vs. MHE Unstable | 0.02* |
|  | MHE-ICT | 0.08 |  |  |
|  | MHE-Stroop | 0.57 | MHE Stable vs. MHE Developed | 0.006* |
|  | MHE-ALL-tests | 0.64 |  |  |

PHES: psychometric hepatic encephalopathy score, ICT: inhibitory control test, MHE: minimal hepatic encephalopathy, All tests: No-MHE is normal on all three, MHE is abnormal on all three

| Cross-sectional P0.05, r>0.60 | **PHES** | | **ICT** | | **Stroop** | | **ALL tests** | |
| --- | --- | --- | --- | --- | --- | --- | --- | --- |
|  | No MHE | MHE | No MHE | MHE | No MHE | MHE | No MHE | MHE |
| Clustering coefficient | 0.312 | 0.257 | 0 | 0 | 0.092 | 0 | - | 0.307 |
| Network centralization | 0.372 | 0.345 | 0.186 | 0.695 | 0.137 | 1 | - | 0.269 |
| Average no of neighbors | 17.953 | 14.041 | 2.576 | 1.875 | 4.506 | 1.8 | - | 21.114 |
| No. of nodes | 170 | 145 | 65 | 49 | 87 | 42 | - | 140 |
| Network density | 0.106 | 0.098 | 0.044 | 0.125 | 0.052 | 0.2 | - | 0.152 |
| Network heterogeneity | 0.869 | 0.883 | 1.031 | 1.277 | 0.836 | 1.333 | - | 0.681 |

Table S2: Correlation network characteristics

PHES: psychometric hepatic encephalopathy score, ICT: inhibitory control test, MHE: minimal hepatic encephalopathy, All tests: No-MHE is normal on all three, MHE is abnormal on all three

Table S3: Clinical Characteristics and Alpha Diversity Measures in Longitudinal Analyses

|  | No-MHE Stable  (n=8 pairs) | | MHE Stable  (n=20 pairs) | | Developed MHE (n=5 pairs) | | Resolved MHE  (n=4 pairs) | |
| --- | --- | --- | --- | --- | --- | --- | --- | --- |
| Mean+/-SD | Pre | Post | Pre | Post | Pre | Post | Pre | Post |
| MELD | 8.0±2.0 | 8.8±1.8 | 12.6±3.1 | 12.4±3.4 | 12.3±4.6 | 13.0±5.4 | 10.0±3.0 | 12.0±3.8 |
| PPI | 5 | 6 | 17 | 17 | 2 | 2 | 2 | 2 |
| Lactulose | 3 | 6 | 19 | 20 | 1 | 3 | 3 | 3 |
| Rifaximin | 3 | 6 | 18 | 19 | 0 | 2 | 3 | 3 |
| Prior HE | 3 | 5 | 19 | 20 | 1 | 4 | 3 | 3 |
| Ascites worsening | - | 2 | - | 0 | - | 3 | - | 0 |
| HE episodes | - | 3 | - | 0 | - | 3 | - | 0 |
| SBP | - | 0 | - | 1 | - | 1 | - | 0 |
| **Alpha bacterial diversity** | | | | | | |  |  |
| Shannon | 2.7±0.6 | 2.6±0.3 | 2.2±0.9 | 2.3±0.6 | 2.8±0.2 | 2.8±0.3 | 2.5±0.6 | 2.8±0.6 |
| Simpson | 0.9±0.1 | 0.9±0.1 | 0.7±0.2 | 0.8±0.1 | 0.9±0.00 | 0.9±0.00 | 0.8±0.1 | 0.9±0.1 |
| Chao1 | 80.0±18.3 | 79.0±13.9 | 75.1±17.2 | 76.3±16.5 | 90.0±19.6 | 81.6±18.6 | 93.5±18.1 | 79.8±25.6 |
| **Alpha viral diversity** | | | | | | |  |  |
| Shannon | 0.9±0.6 | 1.2±0.7 | 1.2±0.5 | 1.1±0.6 | 1.1±0.6 | 1.1±0.7 | 1.9±0.6 | 0.9±0.7 |
| Simpson | 0.4±0.3 | 0.5±0.3 | 0.5±0.2 | 0.5±0.2 | 0.5±0.2 | 0.5±0.3 | 0.7±0.1 | 0.3±0.3 |
| Chao1 | 14.0±10.3 | 18.1±9.2 | 17.8±7.5 | 15.3±6.6 | 14.0±14.6 | 15.8±9.2 | 27.0±14.2 | 14.3±8.5 |

MHE: minimal hepatic encephalopathy, MHE was defined using PHES (psychometric hepatic encephalopathy score), SBP: spontaneous bacterial peritonitis, MELD: model for end-stage liver disease score, PPI: proton pump inhibitors.

Table S4: Clinical and LEFse changes between all stable (MHE+No-MHE) and all unstable (developed MHE or resolved MHE) patients

| Clinical parameters | All Stable (n=27 pairs) | | All unstable (n=9 pairs) | | LEFSe | All Stable  (n=27 pairs) | All unstable  (n=9 pairs) |
| --- | --- | --- | --- | --- | --- | --- | --- |
| Mean+/-SD | Pre | Post | Pre | Post | **Bacteria significant**  **LDA score > 3** | *Bifidobacterium pseudo*  *catenulatum*  *Lactobacillus casei paracasei*  *Bifidobacterium adolescentis*  *Bifidobacterium breve*  *Bacteroides vulgatus* | *Bacteroides uniformis*  *Bacteroides faecis*  *Haemophilus parainfluenzae*  *Enterobacter cloacae*  *Roseburia inulinivorans*  *Prevotella melaninogenica*  *Ruminococcus gnavus*  *Actinobacillus unclassified*  *Odoribacter splanchnicus*  *Ruminococcus_sp_5_1_39BFAA*  *Bacteroides_sp_3_1_19*  *Eubacterium eligens* |
| MELD score | 11.25±3.44 | 11.32±3.43 | 11.00±3.94 | 12.56±4.48 |  |  |  |
| PPI use | 22 | 23 | 4 | 4 |  |  |  |
| Lactulose use | 22 | 26 | 4 | 6 |  |  |  |
| Rifaximin use | 21 | 25 | 3 | 5 |  |  |  |
| Overt HE history | 22 | 26 | 4 | 7 |  |  |  |
| Bacterial diversity |  |  |  |  |  |  |  |
| Shannon | 2.4±0.8 | 2.4±0.5 | 2.6±0.4 | 2.8±0.4 | **Viruses significant**  **LDA score > 2** | N/A | *Streptococcus phageJavan349*  *Streptococcus phageJavan348*  *Streptococcus satellite phageJavan323*  *Faecalibacterium phage FP_Brigit*  *Streptococcus satellite phage Javan378*  *Streptococcus phage Javan336*  *Streptococcus phage Javan371*  *Lactococcus phage CHPC971*  *Streptococcus phageJavan366*  *Streptococcus phageJavan268*  *Streptococcus phageJavan266*  *Escherichia virus If1*  *Streptococcus satellite phageJavan305* |
| Simpson | 0.8±0.2 | 0.8±0.1 | 0.9±0.1 | 0.9±0.0 |  |  |  |
| Chao1 | 76.6±17.3 | 77.1±15.6 | 91.6±17.9 | 80.8±20.4 |  |  |  |
| Viral diversity |  |  |  |  |  |  |  |
| Shannon | 1.1±0.5 | 1.1±0.6 | 1.5±0.7 | 1.0±0.7 |  |  |  |
| Simpson | 0.5±0.2 | 0.5±0.3 | 0.6±0.2 | 0.4±0.3 |  |  |  |
| Chao1 | 16.7±8.4 | 16.1±7.4 | 19.8±15.1 | 15.1±8.4 |  |  |  |

Table S5: LEFse results stable No-MHE and resolved MHE

| Longitudinal MHE-PHES | **No-MHE_Stable : no changes** (n=8 pairs ) | **Resolved MHE** (n=4 pairs ) |
| --- | --- | --- |
| **Bacteria significant**  **LDA score > 3** | *Streptococcus_salivarius*  *Ruminococcus_obeum*  *Coprococcus_comes*  *Bacteroides_xylanisolvens* | *Bacteroides_uniformis*  *Bifidobacterium_dentium*  *Ruminococcus_gnavus*  *Escherichia_unclassified*  *Bacteroides_sp_4_3_47FAA*  *Clostridium_perfringens*  *Klebsiella_pneumoniae*  *Bacteroides_sp_3_1_19* |
| **Viruses significant**  **LDA score > 2** | *N/A* | *Streptococcus phageJavan349*  *Streptococcus phageJavan348*  *Streptococcus satellite phageJavan323*  *Streptococcus phage Javan278*  *Streptococcus satellite phage Javan378*  *Streptococcus phage Javan371*  *Streptococcus phage Javan326*  *Streptococcus phage Javan266*  *EscherichiavirusIf1*  *GokushovirusWZ_2015a*  *Streptococcus satellite phageJavan305* |

**Supplementary figure legends**

**Figure S1.** LefSe analysis in MHE vs. No-MHE in cross-sectional study of bacteria (in left panel) and viruses (in right panel) of PHES (A), ICT (B), Stroop (C), All test (D)

**Figure S2.** LefSe analysis in MHE vs. No-MHE in longitudinal study of bacteria (in upper panel) and viruses (in lower panel) of Stable vs. unstable (A,C), and Stable MHE vs. developed MHE (B,D)

**Supplementary references:**

1. Allampati S, Duarte-Rojo A, Thacker LR, Patidar KR, White MB, Klair JS, et al. Diagnosis of Minimal Hepatic Encephalopathy Using Stroop EncephalApp: A Multicenter US-Based, Norm-Based Study. Am J Gastroenterol 2016; 111:78-86.

2. Vilstrup H, Amodio P, Bajaj J, Cordoba J, Ferenci P, Mullen KD, et al. Hepatic encephalopathy in chronic liver disease: 2014 Practice Guideline by the American Association for the Study of Liver Diseases and the European Association for the Study of the Liver. Hepatology 2014; 60:715-35.

3. Weissenborn K, Ennen JC, Schomerus H, Ruckert N, Hecker H. Neuropsychological characterization of hepatic encephalopathy. J Hepatol 2001; 34:768-73.

4. Bajaj JS, Saeian K, Verber MD, Hischke D, Hoffmann RG, Franco J, et al. Inhibitory control test is a simple method to diagnose minimal hepatic encephalopathy and predict development of overt hepatic encephalopathy. Am J Gastroenterol 2007; 102:754-60.

5. Bajaj JS, Heuman DM, Sterling RK, Sanyal AJ, Siddiqui M, Matherly S, et al. Validation of EncephalApp, Smartphone-Based Stroop Test, for the Diagnosis of Covert Hepatic Encephalopathy. Clin Gastroenterol Hepatol 2014.
