# Supplementary material for: Alterations in gut virome are associated with cognitive function and minimal hepatic encephalopathy cross-sectionally and longitudinally in cirrhosis
Source: Gut Microbes. 2023 Nov 27;15(2):2288168. doi: 10.1080/19490976.2023.2288168 (PMC10730154; doi:10.1080/19490976.2023.2288168)
Supplement: Gut Microbes Supplementary Figures_110823.docx [file KGMI_A_2288168_SM6797.docx]

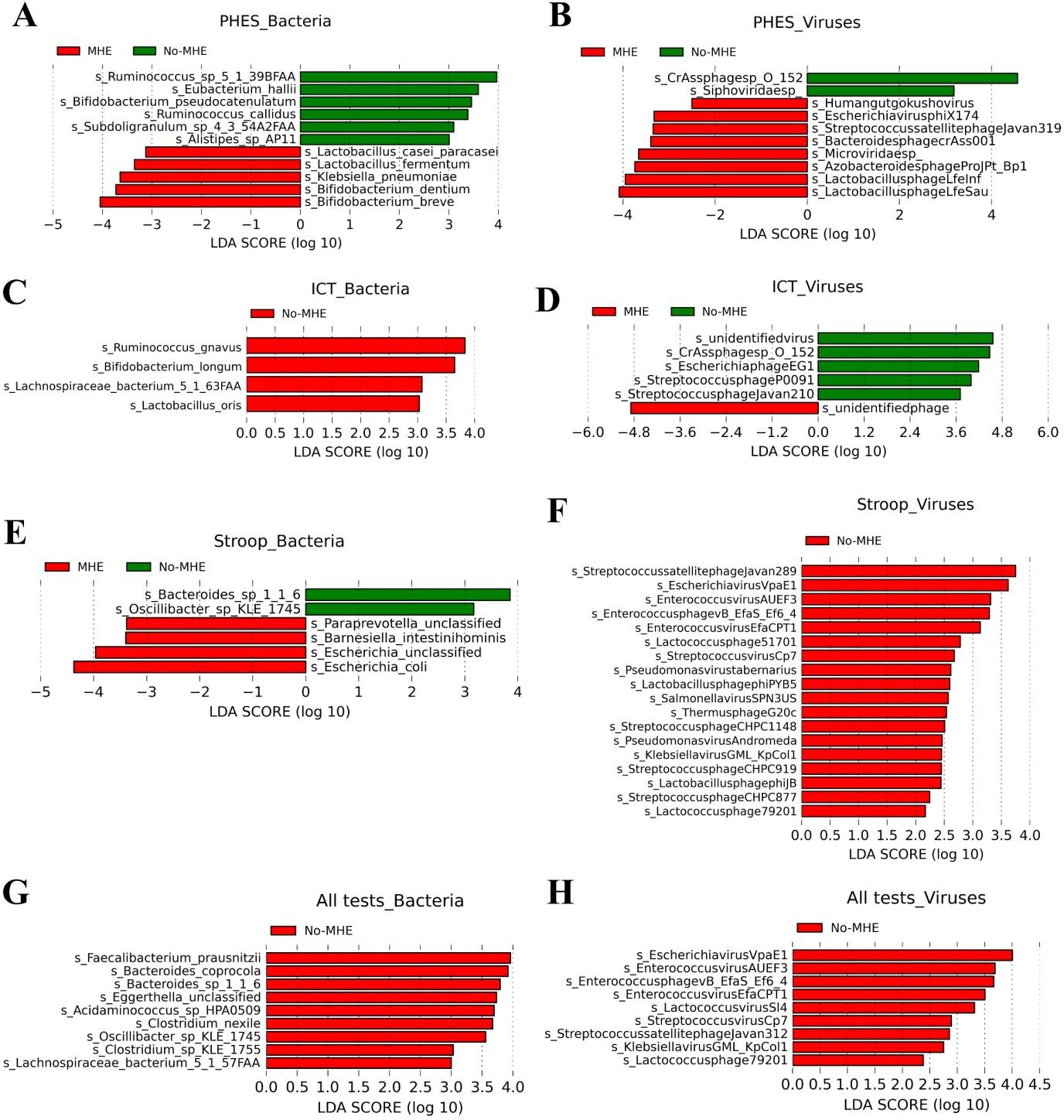


**Figure S1.** LEfSe analysis in MHE vs. No-MHE in cross-sectional study of bacteria (in left panel) and viruses (in right panel) of PHES (A), ICT (B) , Stroop (C) , All test (D)


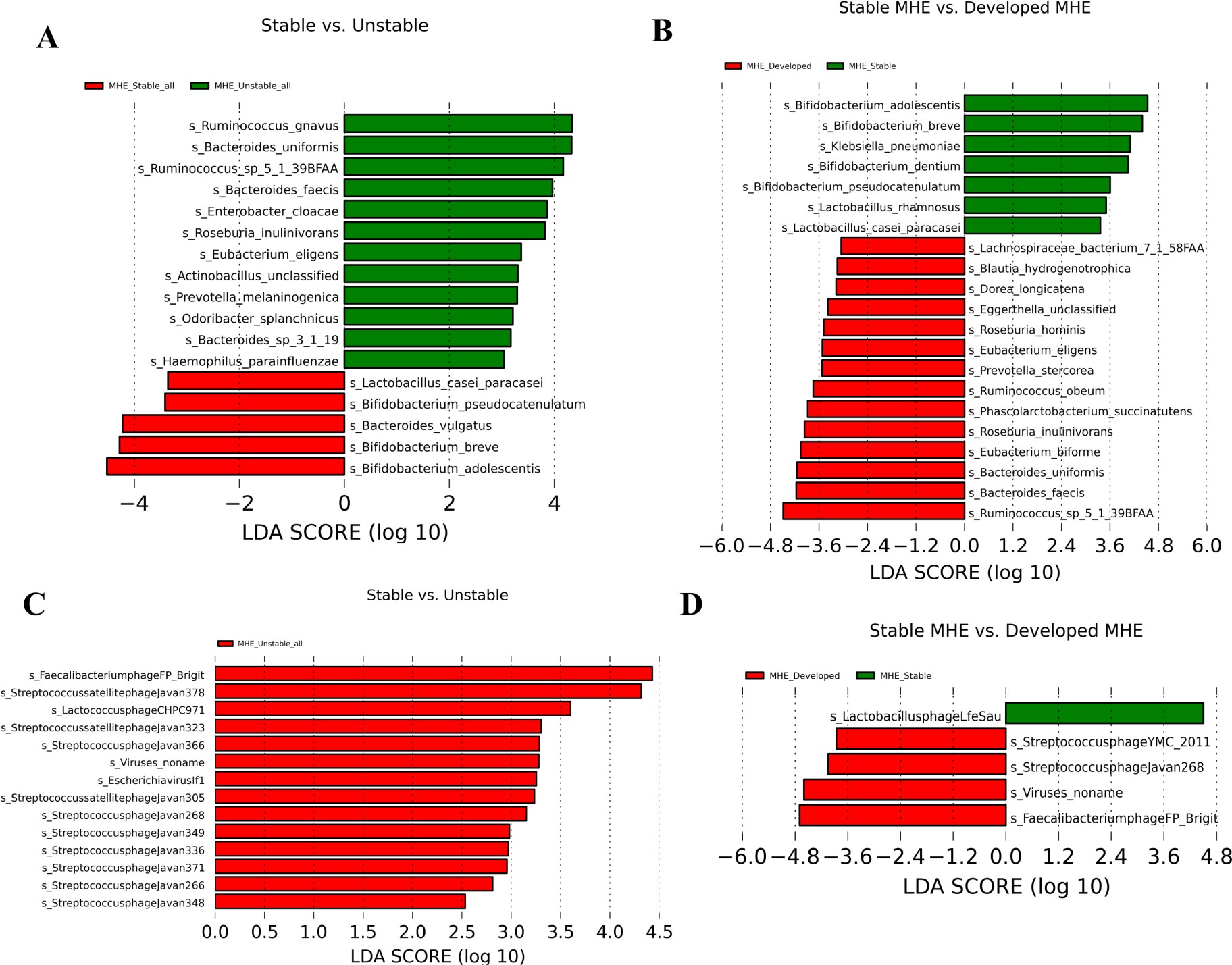


**Figure S2.** LEfSe analysis in MHE vs. No-MHE in longitudinal study of bacteria (in upper panel) and viruses (in lower panel) of Stable vs. unstable (A,C), and Stable MHE vs. developed MHE (B,D)
